# Why so stressed? A comparative study on stressors and stress between hospital and non-hospital nurses

**DOI:** 10.1186/s12912-020-00511-0

**Published:** 2021-01-04

**Authors:** Rosnawati Muhamad Robat, Mohd Fadhli Mohd Fauzi, Nur Adibah Mat Saruan, Hanizah Mohd Yusoff, Abdul Aziz Harith

**Affiliations:** 1 Occupational and Environmental Health Unit, Selangor State Health Department, No 1 Wisma Sunway, Jalan Tengku Ampuan Zabedah C 9/C, Seksyen 9, 40100 Shah Alam, Selangor Malaysia; 2 Ministry of Health Malaysia, Block E1, E3, E6, E7 & E10, Complex E, Federal Government Administrative Centre, 62590 Putrajaya, Malaysia; 3 Department of Community Health, Faculty of Medicine, Universiti Kebangsaan Malaysia, Jalan Yaacob Latiff, Bandar Tun Razak, 56000 Kuala Lumpur, Malaysia; 4 Occupational Health Research Centre, Institute for Public Health Malaysia, Blok B5 & B6, Kompleks NIH, No1, Jalan Setia Murni U13/52, Seksyen U13 Bandar Setia Alam, 40170 Shah Alam, Selangor Malaysia

**Keywords:** Stress, Stressor, Workplace, Household, Nurse, Hospital, Shift work

## Abstract

**Background:**

Stress, which can be attributed to household and workplace stressors, is prevalent among nurses. However, these stressors’ attribution may differ between hospital and non-hospital nurses. It is currently unknown whether there are significant differences in the sociodemographic and occupational characteristics between hospital and non-hospital nurses which may potentially influence the type and magnitude of stressors, and subsequently the stress status. Therefore, this study aims to estimate the prevalence of stress and compare the roles of sociodemograhic characteristics, occupational profiles, workplace stressors and household stressors in determining the stress status between hospital and non-hospital female nurses in Malaysia.

**Methods:**

This cross-sectional study was conducted among randomly-selected 715 female nurses in Malaysia using pencil-and-paper self-reported questionnaires.

**Results:**

The majority of participants were ever married (87.0%), having children (76.2%), and work in hospital setting (64.8%). The level of household stressors was generally similar between hospital and non-hospital nurses. However, hospital nurses significantly perceived higher level of workplace stressors. Shift work is significantly associated with higher level of household and workplace stressors among nurses in both groups. The level of stress was significantly higher among hospital nurses. Both household and workplace stressors explained about 40% of stress status in both hospital and non-hospital nurses.

**Conclusion:**

Hospital nurses are at higher risk of having stressors and stress as compared to non-hospital nurses, probably due to higher proportion of them involved in shift work. Hospital nurses should be given high priority in mitigating stress among nurses.

## Background

Nurses play a vital role in Malaysia’s healthcare system and are an essential part of medical workforce [1]. The nursing profession is regarded as one of the most demanding and stressful occupations [2]. Within their occupation, nurses are exposed to physical, mental, temporal, and emotional demands which exert sustained physical and psychological effort [3–5]. Nurses, who are mostly women [6], are also more likely to be exposed to household/family demands such as childcare and household chores [7]. Both workplace and household demands results in a build-up stressors which are associated with physiological and/or psychological burden, which consequently contributes to increased stress [3, 8].

Stress is a psychological result of an imbalance between perceptions of external demands and the internal resources available to meet those demands [9]. In other words, the workplace or household demands are not necessarily the causes of stress; they become stressors if they cause excessive exertion which is then not followed by adequate recovery due to poor resilience or ineffective coping strategies [9, 10]. A recent study among Australian nurses reported a 41.2% stress prevalence; with 24.51%, 10.8% and 5.88% being categorized as mild/moderate, severe, and extremely severe stress, respectively [11]. Among the predictive factors of stress include job satisfaction [11], high workloads [12], shift work [13], sleep quality [13], and nurse’s practice environment [14]. Unmanaged stress can be harmful to a nurse’s health leading to unwanted consequences such as burnout [15] and work performance issues such as absenteeism or presenteeism [16].

This current study focusses on female nurses working at government health facilities because workplace stress is more likely among the public service employee [17], while household stress is more common among women [7]. Women, as compared to men, are at a higher risk of having stress as they perceive stressors to be more threatening [18–20]. Female nurses as working women take on multiple roles simultaneously in their daily life; these include the important roles at home as a mother involved in childcare, as a wife, as an informal care-giver to family members who need help such as parents, as a breadwinner supporting the financial needs of herself and/or her family, and as a house member who is mutually responsible to do household chores as well as ensuring safety of all members [21–24]. The roles she plays in her occupation include nursing role such as documentation, education, medication administration, patient care, and communication, employee roles who plays a vital act in achieving the organization’s goal, and roles as a colleague who part of a team with other nurses, doctors, and other staff [25, 26]. In general, having multiple roles can create work-home and role conflict, leading to an increased risk of psychological distress [21, 24].

Although given the same job title ‘nurse’, stressors differ vastly between those employed in the hospital setting versus those who aren’t. Such an instance is the shift system employed by hospital nurses. This shift system has the consequence of limiting time spent with family members which can be a cause of conflict with them [27, 28]. It can also trigger contention with fellow doctors and nurses during pass-over session [27, 28]. Nurses in hospital setting also have to handle the high workload related to the care of acute patients with complicated medical procedures, and occasionally, they have to handle events related to the death of these patients [27–29]. In contrast, nurses at non-hospital setting which typically operate in non-shift schedule may have better staff support and less workplace conflict as they are more likely to work in a same team every day [30]. They may also have better preparation to deal with their work as their work is more likely involve non-acute patients and patient with long-term follow-up [30]. Due to the non-acute nature of patients, non-hospital nurses also have higher autonomy as they are less likely to communicate their findings to the doctors [30, 31]. With an exception of one study [28], most comparisons between hospital and non-hospital nurses described above were not statistically examined.

Although the roles of stressors towards stress among nurses have generally been widely established, there are limited studies that analytically compare their relationship in different work conditions, particularly hospital and non-hospital settings among nurses. It is unknown whether there are significant differences in the sociodemographic and occupational characteristics between both groups. As the background characteristic may potentially influence the type and magnitude of stressors, and subsequently the stress status, it is also unknown whether stressors and stress status are significantly differing between hospital and non-hospital nurses. The establishment of evidence on these differences is important to support the need of targeted intervention which may differ between the workplace setting. Therefore, this study aims to examine and compare the sociodemograhic and occupational profiles, workplace and household stressors, and the stress status between hospital and non-hospital nurses. This study also aims to identify and compare the roles of sociodemograhic characteristics, occupational profiles, workplace stressors and household stressors in determining the stress status between both groups.

## Methods

### Study design and sampling

This is a comparative analytic cross-sectional study conducted in year 2018 among registered nurses working at all government health facilities in the state of Selangor, Malaysia. The inclusion criteria are all registered Malaysian female nurses from various position including matrons, sisters, staff nurses, midwives and community nurses who have been working at their current workplace for at least 6 months. Matrons and sisters are categorized as nurse managers; a matron is responsible in ensuring the smooth function of respective departments, hospitals, or districts, while a sister is responsible in administration of patient care in respective patient care unit such as ward or clinic. Staff nurses, midwives and community nurses can be classified as implementers; a staff nurse and a midwife are responsible to provide individualized care for all patients in their respective patient care units, while community nurses are responsible to assist staff nurses and midwifes in the delivery of patient care. Those who are medically-diagnosed as having psychiatric illnesses or on psychiatric medication were excluded. Eligible nurses’ name was randomly chosen by using Microsoft Excel software. Based on the stress prevalence of 0.25 [32, 33] and 0.49 [34] among Malaysian nurses, precision of 0.05, and power of 0.8, the minimum sample size required was 289 and 385. Since this is a comparative cross-sectional study, the sample size was doubled to 770 nurses.

### Study instruments

Data on participants’ sociodemographic and occupational profile were collected using a self-reported questionnaire containing 18 items that inquire on the age, marital status, number of children, work tenure, job position, workplace, schedule system and others (Additional file 1).

Stress status was measured by using validated Malay version of Personal Stress Inventory: Sign and Symptoms of Stress questionnaire [35, 36] by asking the frequency of signs and symptoms of stress experienced by the participants. The inventory consisted of 51 items with 11 subscales using a four-point Likert scale i.e. ‘never’ (0), ‘once or twice’ (1), ‘every week’ (2) and ‘nearly every day’ (3). A total score was obtained by adding the nurse’s responses to all 51 items, ranging from 0 to 153. Those who scored ≥36 were classified as stressed. Numerically, a higher score indicates a higher level of stress. The Cronbach alpha of this instrument is 0.968.

Household stressors were measured by using validated Malay version of Personal Stress Inventory: Pressures and Demands from Family and Household [35, 36]. This is a brief instrument that assesses the degree to which the situation in a family and household perceived as a stressor for the respondent. The inventory consisted of 12 items which included ‘not enough money’, ‘conflict with spouse’, ‘conflicts over household tasks’, ‘problems or conflict with children’, ‘pressure from relatives or in-laws’, ‘fixing up of the house’, ‘not enough time to spend with family’, ‘sexual conflict or frustration’, ‘dangerous or stressful surroundings and neighbourhood’, ‘conflict with close friend or relatives’, ‘personal problem causing strain in family’ and ‘no babysitter’. This questionnaire used a four-point Likert-type scale i.e. ‘none at all’ (0), ‘a little’ (1), ‘some’ (2) and ‘a great deal’ (3). A total score was obtained by adding the nurse’s responses to all 12 questions. A total score ranged from 0 to 36. Higher scores indicated a higher level of household stressors. The Cronbach alpha of this instrument is 0.875.

Workplace stressors were measured by using a validated Malay version of Nursing Stress Scale (NSS) [37, 38]. It measures the perceived frequency of the occurrence of stress in the nursing environment. The scale consisted of 34 items with seven subscales, namely ‘workload’ (α = 0.808), ‘dealing with death and dying’ (α = 0.856), ‘conflict with doctors’ (α = 0.798), ‘uncertainty concerning treatment’ (α = 0.844), ‘lack of staff support’ (α = 0.865), ‘conflict with other nurses or supervisors’ (α = 0.759) and ‘inadequate preparation to deal with emotional needs of the patients and their families’ (α = 0.838). The Cronbach alpha of this instrument is 0.832. The NSS was scored on a four-point Likert-type scale from ‘never’ (0), ‘occasionally’ (1), ‘frequently’ (2) to ‘very frequently’ (3). All items were about potentially stressful situations in the nursing workplace. Scoring was conducted by adding up the individual item responses for each subscale. High scores indicated the frequent presence of a specific source of stress. Overall score was determined by adding up all 34-item responses. The total score represented the overall frequency of stress as experienced by a nurse which ranged from 0 to 102.

### Data collection

Participants were approached at their workplace and were given explanation on this study. They were given adequate time of about 1 week to make decision. If they agreed to participate, they were given a set of questionnaires. They were given another day and up to 3 days, to complete the questionnaires.

### Data analysis

Data was initially analyzed descriptively to demonstrate the representativeness of participants involved in this study. Bivariable analysis was then conducted to compare the sociodemographic and occupational characteristic, stressors profile, and stress status/level between the two groups. Next, multiple linear regression using enter method was conducted to determine the significant determinants of stressors for hospital and non-hospital nurses. Hierarchical regression was then conducted in four steps to determine the determinants associated with stress level. In the first step, sociodemographic variables i.e. age, marital status and children were entered. In the second step, occupational variables i.e. work tenure, job position, and work schedule were entered. In the third step, both workplace and household stressors were entered. In the fourth step, interaction term between workplace and household stressors were entered. Statistically significant result was set at *p* < 0.05.

## Results

### Participants characteristic

The response rate was 92.9% (*n* = 715). Participants were generally in middle aged with a mean age of 34.63 (SD = 8.050) years and a mean work tenure of 11.40 (SD = 7.461) years. The majority of participants were married (87.0%) and have at least one child (76.2%). Most of them hold a position as community nurse or staff nurse (85.0%), work in hospital setting (64.8%) and involved in shift schedule (64.8%).

### Group comparisons

Table 1 and Table 2 demonstrates the comparison of sociodemographic and occupational profiles, workplace and household stressors, and stress status between hospital and non-hospital nurses. There was no significant difference in the sociodemographic and occupational profiles of the two groups except for work schedule and job position. The proportion of participants working in shift and holding a job position as a community nurse or a staff nurse was significantly higher among hospital nurses as compared to non-hospital nurses. There was no significant difference in the overall score of household stressor between hospital and non-hospital nurses. However, hospital nurses had significantly higher level of household stressors related to ‘not enough money’, ‘no time with family’ and ‘personal problem cause strain’. With regards to workplace stressors, hospital nurses had significantly higher overall score of workplace stressors and each of its components namely ‘workload’, ‘death and dying’, ‘inadequate preparation’, lack of staff support’, ‘uncertain treatment’, ‘conflict with doctors’, and ‘conflict with nurses’. The overall prevalence of stress among participants was 27.3%. Although the hospital nurses had significantly higher level of stress score as compared to non-hospital nurses, there is no significant difference in the prevalence of stress between both groups.

**Table 1:** Comparison of numerical variables using Student’s T-test

Variables	Mean (SD)			Mean Difference	95% CI	t	df	p
	Total (n = 715)	Non-Hospital (n = 252)	Hospital (n = 463)					
**Sociodemographic profile**								
Age, in years	34.63 (8.050)	34.42 (7.718)	34.74 (8.232)	−0.32	− 1.56, 0.92	− 0.508	713	0.61
No. of children	1.87 (1.497)	1.84 (1.464)	1.88 (1.516)	−0.04	− 0.27, 0.19	− 0.322	713	0.75
Work tenure, in years	11.40 (7.461)	11.11 (7.364)	11.56 (7.517)	−0.45	−1.60, 0.70	− 0.767	713	0.44
**Household stressors**	**5.91 (5.468)**	**5.71 (5.747)**	**6.02 (5.314)**	**−0.31**	**− 1.15, 0.53**	**− 0.727**	**713**	**0.47**
Not enough money	0.68 (0.794)	0.62 (0.803)	0.72 (0.788)	−0.11	− 0.23, 0.02	−1.712	713	0.09
Conflict with spouse	0.49 (0.689)	0.48 (0.676)	0.49 (0.696)	−0.02	− 0.12, 0.09	− 0.301	713	0.76
Conflict over household task	0.48 (0.678)	0.45 (0.675)	0.50 (0.680)	−0.05	− 0.16, 0.05	− 0.992	713	0.32
Conflict with children	0.37 (0.596)	0.41 (0.653)	0.34 (0.562)	0.07	−0.02, 0.17	1.511	453.79	0.13
Pressure from relatives	0.44 (0.712)	0.49 (0.770)	0.42 (0.678)	0.07	−0.04, 0.18	1.231	462.73	0.22
Fixing up house	0.42 (0.672)	0.48 (0.770)	0.39 (0.611)	0.09	−0.03, 0.20	1.517	425.11	0.13
No time with family	1.08 (0.961)	0.98 (0.972)	1.13 (0.951)	−0.15	−0.30, 0.00	−1.991	713	0.05
Sexual conflict	0.21 (0.510)	0.23 (0.560)	0.20 (0.482)	0.03	−0.04, 0.11	0.841	713	0.40
Dangerous surroundings	0.43 (0.675)	0.41 (0.683)	0.44 (0.671)	−0.02	−0.13, 0.08	− 0.446	713	0.66
Conflict with close friends	0.40 (0.607)	0.36 (0.578)	0.42 (0.622)	−0.06	− 0.16, 0.03	−1.302	713	0.19
Personal problem cause strain	0.41 (0.631)	0.34 (0.559)	0.44 (0.665)	−0.11	−0.20, − 0.01	−2.252	595.07	0.02
No babysitter	0.51 (0.793)	0.48 (0.733)	0.54 (0.823)	−0.06	−0.18, 0.06	− 0.958	713	0.34
**Workplace stressors**	**25.86 (13.384)**	**20.85 (11.983)**	**28.59 (13.330)**	**−7.74**	**−9.66, −5.82**	**−7.924**	**563.98**	**0.00**
Workload	8.36 (3.593)	7.63 (3.468)	8.75 (3.601)	−1.13	−1.67, − 0.58	−4.049	713	0.00
Death and dying	4.35 (3.784)	2.44 (2.706)	5.39 (3.882)	−2.95	−3.44, −2.47	−11.896	670.90	0.00
Inadequate preparation	1.79 (1.570)	1.35 (1.405)	2.02 (1.605)	−0.67	− 0.91, − 0.43	−5.570	713	0.00
Lack of staff support	2.12 (1.895)	1.70 (1.734)	2.35 (1.941)	−0.65	−0.93, − 0.36	−4.406	713	0.00
Uncertain treatment	3.20 (2.418)	2.51 (2.354)	3.57 (2.372)	−1.06	−1.42, −0.69	−5.714	713	0.00
Conflict with doctors	3.24 (2.493)	2.75 (2.412)	3.50 (2.499)	−0.76	−1.14, − 0.38	−3.918	713	0.00
Conflict with nurses	2.81 (2.454)	2.47 (2.536)	3.00 (2.390)	−0.53	−0.90, − 0.15	−2.758	713	0.01
**Stress score**	**25.47 (20.704)**	**22.04 (18.472)**	**27.34 (21.613)**	**−5.31**	**−8.47, −2.15**	**−3.296**	**713**	**0.00**

**Table 2:** Comparison of categorical variables using chi square test

	n (%)^a^	n (%)^b^		χ^**2**^	df	p
	Total (n = 715)	Non-Hospital (n = 252)	Hospital (n = 463)			
**Marital status**						
Never married	93 (13.0)	30 (32.3)	63 (67.7)	0.418	1	0.518
Ever married	622 (87.0)	222 (35.7)	400 (64.3)			
**Having children**						
None	170 (23.8)	55 (32.4)	115 (67.6)	0.817	1	0.366
At least one	545 (76.2)	197 (36.1)	348 (63.9)			
**Work tenure**						
Less than 10 years	332 (46.4)	123 (37.0)	209 (63.0)	0.883	1	0.347
10 years and above	383 (53.6)	129 (33.7)	254 (66.3)			
**Work schedule**						
Non-shift	252 (35.2)	176 (69.8)	76 (30.2)	204.090	1	< 0.001
Shift	463 (64.8)	76 (16.4)	387 (83.6)			
**Position**						
Staff nurse / community nurse	608 (85.0)	229 (37.7)	379 (62.3)	10.423	1	0.001
Nurse manager	107 (15.0)	23 (21.5)	84 (78.5)			
**Stress status**						
Not stress	520 (72.7)	194 (37.3)	326 (62.7)	3.555	1	0.059
Stress	195 (27.3)	58 (29.7)	137 (70.3)			

^a^column percent; ^b^ row percent

### Inter-correlation among the study measures in the two study groups

Table 3 shows the inter-correlation among sociodemographic profiles (i.e. age, children), occupational profiles (i.e. work tenure), stressors (i.e. workplace and household) and stress levels. Age, number of children, and work tenure were negatively correlated with workplace stressors. In contrast, number of children and workplace stressors were positively correlated with household stressors. Both workplace and household stressors were moderately and positively correlated with stress score.

**Table 3:** Correlation among sociodemographic profile, occupational profile, stressors and stress score

Variables	Age	Children	Work tenure	Workplace stressors	Household stressors	Stress score
**Age**	1					
**Children**	.479^a^	1				
**Work tenure**	.949^a^	.481^a^	1			
**Workplace stressors**	−.110^a^	−.090^b^	−.101^a^	1		
**Household stressors**	−0.015	.085^b^	−0.017	.449^a^	1	
**Stress score**	−0.059	−0.034	−.076^b^	.535^a^	.561^a^	1

^a^ Correlation is significant at the 0.01 level (2-tailed); ^b^ Correlation is significant at the 0.05 level (2-tailed)

### Linear regression analysis predicting stressors among the two study groups

Table 4 demonstrates the determinants of household and workplace stressors. Marriage is associated with higher household stressors regardless of workplace. In contrast, job position of nurse manager and involvement in shift schedule are associated with higher workplace stressors only among hospital nurses. Shift work is also associated with higher household stressors only among hospital nurses.

**Table 4:** Multiple linear regression to identify determinants of stressors

Variables	Adj. β (95%CI)					
	Household Stressors			Workplace Stressors		
	All (n = 715)	Non-Hospital (n = 252)	Hospital (n = 463)	All (n = 715)	Non-Hospital (n = 252)	Hospital (n = 463)
**Age**	−0.029 (− 0.187, 0.129)	0.041 (− 0.257, 0.340)	−0.052 (− 0.239, 0.136)	−0.205 (− 0.577, 0.167)	−0.132 (− 0.756, 0.493)	−0.194 (− 0.652, 0.264)
**Marital status**^a^	2.250 (0.905, 3.595) ***	2.932 (0.421, 5.444) *	1.883 (0.280, 3.485) *	0.585 (−2.581, 3.752)	−1.222 (−6.479, 4.036)	1.219 (−2.696, 5.134)
**No. of children**	0.266 (−0.057, 0.589)	0.154 (−0.431, 0.738)	0.319 (− 0.618, 0.707)	−0.384 (−1.145, 0.377)	0.227 (− 0.996, 1.449)	−0.791 (− 1.737, 0.156)
**Work tenure**	− 0.031 (− 0.204, 0.142)	−0.009 (− 0.319, 0.300)	−0.050 (− 0.262, 0.163)	−0.049 (− 0.457, 0.359)	−0.200 (− 0.848, 0.447)	0.046 (− 0.473, 0.565)
**Job position**^b^	−0.122 (− 1.497, 1.254)	−1.589 (−4.571, 1.393)	0.510 (− 1.056, 2.076)	6.310 (3.070, 9.550) ***	1.730 (−4.511, 7.971)	6.086 (2.260, 9.912) **
**Work schedule**^**c**^	0.896 (0.056, 1.737) *	0.316 (−1.237, 1.869)	1.370 (0.020, 2.719) *	7.470 (5.491, 9.449) ***	0.759 (−2.491, 4.010)	8.647 (5.349, 11.944) ***
**R**^**2**^	0.024	0.020	0.026	0.097	0.012	0.076

*** *p* < 0.001 (2-tailed); ** *p* < 0.01 (2-tailed); * *p* < 0.05 (2-tailed); ^a^ Marital status (0 = never married, 1 = ever married); ^b^ Job position (0 = staff nurse/community nurse, 1 = nurse manager); ^c^ Work schedule (0 = non-shift, 1 = shift)

### Hierarchical regression analysis predicting stress among the two study groups

Table 5 demonstrates the hierarchical linear regression analysis which aims to identify the determinants of stress level among hospital and non-hospital nurses. It was found that workplace and household stressors significantly explained about 38% to 40% variance of the stress level of all participants regardless of their workplace. The stress level is higher among those non-hospital nurses who are of older age, and those who were junior, with concurrent higher magnitude of workplace and household stressors. In contrast, the stress level is higher among nurse managers in hospital settings and those hospital nurses who had higher magnitudes of workplace and household stressors. The interaction between workplace and household stressors did not significantly influence the stress level.

**Table 5:** Hierarchical linear regression to identify determinants of stress level

Variables	All (n = 715)		Non-Hospital (***n*** = 252)		Hospital (***n*** = 463)	
	β in step … ^a^	Final β ^b^	β in step … ^a^	Final β ^b^	β in step … ^a^	Final β ^b^
**Step 1**						
Age	−0.157 (− 0.375, 0.062)	0.477 (0.014, 0.939) *	0.014 (− 0.326, 0.354)	1.074 (0.337, 1.812) **	− 0.249 (− 0.527, 0.030)	0.212 (− 0.381, 0.804)
Marital status^c^	1.966 (−3.168, 7.100)	−1.300 (−5.267, 2.667)	7.537 (− 0.436, 15.511)	3.138 (−3.188, 9.464)	−0.271 (− 6.828., 6.286)	−3.441 (−8.528, 1.646)
Having children^d^	− 0.268 (− 1.500, 0.964)	−0.327 (− 1.276, 0.622)	−0.710 (− 2.590, 1.169)	−0.823 (− 2.267, 0.620)	−0.086 (− 1.672, 1.500)	−0.106 (− 1.343, 1.130)
Δ R^2^	0.004		0.014		0.010	
Δ F	1.026		1.209		1.530	
**Step 2**						
Work tenure	−0.741 (− 1.396, − 0.085) *	−0.665 (− 1.172, − 0.158) **	−1.084 (− 2.084, − 0.085) *	−0.978 (− 1.743, − 0.214) *	−0.573 (− 1.435, 0.289)	−0.496 (− 1.167, 0.175)
Job position^e^	7.755 (2.553, 12.957) **	4.644 (0.565, 8.723) *	−1.574 (− 11.204, 8.056)	− 0.128 (−7.542, 7.286)	10.192 (3.840, 16.545) **	6.156 (1.158, 11.155) *
Work schedule^f^	5.250 (2.073, 8.428) ***	−0.057 (− 2.612, 2.497)	− 0.654 (− 5.670, 4.362)	−1.385 (− 5.223, 2.452)	6.556 (1.081, 12.031) *	−0.236 (− 4.617, 4.146)
Δ R^2^	0.031		0.020		0.034	
Δ F	7.562 ***		1.653		5.365 ***	
**Step 3**						
Workplace stressor (WS)	0.522 (0.419, 0.625) ***	0.514 (0.376, 0.652) ***	0.543 (0.370, 0.716) ***	0.678 (0.449, 0.907) ***	0.513 (0.379, 0.647) ***	0.446 (0.263, 0.628) ***
Household stressor (HS)	1.573 (1.330, 1.817) ***	1.531 (1.006, 2.055) ***	1.260 (0.898, 1.622) ***	1.722 (1.091, 2.352) ***	1.725 (1.397, 2.052) ***	1.310 (0.480, 2.139) **
Δ R^2^	0.390		0.401		0.380	
Δ F	239.816 ***		86.347 ***		149.765 ***	
**Step 4**						
WS*HS	0.001 (−0.014, 0.017)	0.001 (−0.014, 0.017)	−0.018 (− 0.039, 0.002)	−0.018 (− 0.039, 0.002)	0.012 (− 0.011, 0.035)	0.012 (− 0.011, 0.035)
Δ R^2^	0.000		0.007		0.001	
Δ F	0.032		23.089		1.147	

*** *p* < 0.001 (2-tailed); ** *p* < 0.01 (2-tailed); * *p* < 0.05 (2-tailed); ^a^ β in step.. = β of the particular step at which the variable initially entered the equation; ^b^ Final β = β in the final (4th step); ^c^ Marital status (0 = never married, 1 = ever married); ^d^ Having children (0 = none, 1 = at least one child); ^e^ Job position (0 = staff nurse/community nurse, 1 = nurse manager); ^f^ Work schedule (0 = non-shift, 1 = shift)

## Discussion

This study aims to compare the stress determinants between hospital and non-hospital nurses. The principal findings are: (1) there is not much difference in household stressors between both groups, (2) hospital nurses had significantly higher levels of workplace stressors, (3) the level of stress is higher amongst hospital nurses, (4) shift work is associated with higher household and workplace stressors among hospital nurses, (5) nurse managers in hospital settings are associated with higher level of workplace stressors and stress, (6) marriage is associated with higher household stressors among nurses in both groups, (7) older age and a junior position are associated with higher stress levels among non-hospital nurses, (8) both workplace and household stressors are significantly associated with stress status with 40% explained variance. Overall, hospital nurses are at a higher risk of having workplace stressors, household stressors, and stress.

The hospital nurses had significantly higher stress level in spite of similar prevalence of stress status. This finding is consistent with the evidence from other geographical regions such as Saudi Arabia [39] and Australia [40] which reported higher stress levels among hospital nurses. It could be due to the higher level of all components of workplace stressors and several aspects of household stressors among hospital nurses in our study which explained 40% of variance in stress level. This is supported by a previous finding which found that stress level was significantly and positively correlated with all components of workplace stressors among nurses [40]. Previous studies also reported that hospital nurses may face higher stressors related to workload, death and dying, and conflict with family members or colleagues [27–29]. The stressors could also be implicated by shift work which could adversely impact social, personal, family and occupational life [41, 42] and made complicated by marriage life [39, 42, 43].

Shift work is significantly associated with higher stress level among nurses; however, the significant association is diminished when workplace setting is considered. This is consistent with study by Lin et al. (2015) that reported higher stress level among nurses who work in shift [44]. The diminishing effect could be due to the differential level in proportion of nurses involved in shift work between the two groups. The nature of round-the-clock nursing work seen in hospitals exposes a higher proportion of hospital nurses to shift work which is associated with stressors and stress. Our study further emphasizes that shift schedule is associated with higher risk of having both household and workplace stressors among hospital nurses. This is supported by a study by Ferri et al. (2016) which concludes that shift work, particularly rotating shift work, is a potential stressor for nurses [45]. This finding implies that those involved in shift schedule, particularly hospital nurses, should be given high priority in stress intervention, and the intervention itself should include evaluation and improvement of shift schedule design. For instance, Lin et al. (2015) reported that 2 days-off after night shift will improve the stress level among nurses who are involved in rotating shift work [44].

We also found that nurse managers in hospital settings are associated with higher level of workplace stressors and stress. This could be due to the heavier workload, inadequate resources, and role conflict in fulfilling the demands of their subordinates and superiors [46, 47]. In contrast, an elder age and junior positions are associated with higher stress levels among non-hospital nurses. This is consistent with findings among community health nurses in China and Saudi Arabia [39, 43] which may be explained by low work ability and overstretched among older workers [48] and lower training or competency among junior workers [43]. Nevertheless, all these postulations need to be confirmed in future studies as previous studies did not conduct a comparative study to enable statistical measurement of significant difference.

Our study strengthens the previously gained knowledge that proves difference in mental health status and its determinants between hospital and non-hospital nurses. For instance, a study by Dor et al. (2018) found that hospital nurses had a significantly higher level of emotional exhaustion and depersonalization as compared to community nurses [28]. Another study by Starc (2018) found that nurses from secondary level of healthcare reported higher level of stressors related to dealing with death, working with difficult patients, exposure to infection, working at night, lack of personnel, and working hours as compared to nurses from primary care [49]. Our findings also suggest that working conditions for nurses are not similar, thus, necessary adjustment to accommodate the demands of hospital and non-hospital work should be carried out to ensure a healthy working condition and lower risk of stress.

The workplace stressors and stress levels are significantly higher among hospital nurses. Thus it is necessary to place a high priority on stress level intervention amongst hospital nurses. Intervention should be initially conducted by identifying the root causes of workplace and household stressors such as shift work which could affect work and family life. Further intervention such as schedule redesign should be initiated, and its efficacy should be tested. Apart from hospital nurses, targeted intervention should also focus on high risk groups such as managerial nurse groups in hospital settings, older workers, and juniors in non-hospital settings. Finally, the intervention should consider both household and workplace stressors as both can significantly influence the level of stress among nurses in both hospital and non-hospital settings. To do so, policy makers should first acknowledge that the stressors and stress among nurses are generally different between hospital and non-hospital nurses. Stress-reduction policies that are specifically tailored to hospital and non-hospital nurses should be introduced. This includes conducting training on coping strategies and resilience against workplace and household stressors, consideration of flexible working arrangements for those with conflicting work-home roles, cultivating a stress-free work environment, ‘active case detection’ of nurses with stress at workplace, and provision of psychological support groups at the workplace.

This study has several limitations. First, the use of self-reported data exposes the results to common method bias [50] and social-desirability bias [51]. However, the use of validated questionnaire and guarantee in anonymity may reduce such biases [50, 51]. Second, this study is limited to female nurses and thus cannot be generalized to male nurses. Thirdly, this study was conducted in Malaysia only and may not represent other geographical regions which have different work systems or social cultures.

## Conclusion

Hospital nurses have a higher perceived level of workplace stressors compared to those not in the hospital setting although there is not much difference in household stressors. They also reported higher level of stress score and higher prevalence of stress compared to non-hospital nurses. More attention should be given to hospital nurses in managing stress, particularly those involved in shift system.

## Supplementary Information

**Additional file 1.**

## Data Availability

The data that support the findings of this study are available from Ministry of Health Malaysia, but restrictions apply to the availability of these data, which were used under license for the current study, and so are not publicly available. Data are however available from the authors upon reasonable request and with permission of Ministry of Health Malaysia.

## Abbreviations

NSS: Nursing Stress Scale
HS: Household stressor
WS: Workplace stressor
