# Supplementary material for: Why so stressed? A comparative study on stressors and stress between hospital and non-hospital nurses
Source: BMC Nurs. 2021 Jan 4;20:2. doi: 10.1186/s12912-020-00511-0 (PMC7780689; doi:10.1186/s12912-020-00511-0)
Supplement: Supplementary file 1 — Additional file 1. [file 12912_2020_511_MOESM1_ESM.docx]

**BAHAGIAN A: PROFIL SOSIODEMOGRAFIK (*PART A: SOCIODEMOGRAPGHIC PROFILE*)**

1. Umur (*Age*)

|  |  | tahun (*years*) |
| --- | --- | --- |

1. Jantina (*Gender*)

|  | Perempuan (*Female*) |  | Lelaki (*Male*) |
| --- | --- | --- | --- |

1. Status Perkahwinan (*Marital Status*)

|  | Bujang (*Single*) |  | Berkahwin (*Married*) |  | Bercerai / Berpisah (*Divorce / Separated*) |
| --- | --- | --- | --- | --- | --- |

1. Bilangan Anak (*Number of Children*)

|  |  |
| --- | --- |

**BAHAGIAN B: PROFIL PEKERJAAN (*PART A: OCCUPATIONAL PROFILE*)**

1. Tempoh Perkhidmatan (*Work Tenure*)

|  |  | tahun (*years*) |
| --- | --- | --- |

1. Tempat Perkhidmatan (*Workplace*)

|  | Hospital (*Hospital*) |  | Bukan hospital (*Non-hospital*): ____________________ |
| --- | --- | --- | --- |

1. Jawatan (*Job Position*)

|  | Jururawat Masyarakat (*Community Nurse*) |  | Bidan Terlatih (*Midwife*) |  | Jururawat Terlatih (*Staff nurse*) |  | Ketua Jururawat (*Sister*) |  | Penyelia Jururawat (*Matron*) |
| --- | --- | --- | --- | --- | --- | --- | --- | --- | --- |
|  |  |  |  |  |  |  |  |  |  |

1. Jadual Kerja (*Work Schedule*)

|  | Syif (*Shift*) |  | Bukan Syif (*Non-shift*) |
| --- | --- | --- | --- |

**BAHAGIAN C: TEKANAN DI RUMAH (*PART C: HOUSEHOLD STRESSOR*)**

**Arahan: Sila nyatakan kekerapan situasi yang disenaraikan di bawah ini yang telah menjadi punca tekanan terhadap anda dalam tempoh sebulan yang lepas.** *(Indicate the degree to which the situation referred to has been source of pressure for you in the past month)***.**

| **0** | **1** | **2** | **3** |
| --- | --- | --- | --- |
| Tiada langsung *(none at all)* | Sedikit *(a little)* | Sederhana *(some)* | Banyak *(a great deal)* |

| 1. Tidak cukup wang (*Not enough money)* | **0** | **1** | **2** | **3** |
| --- | --- | --- | --- | --- |
| 2. Konflik dengan suami (*Conflicts with spouse)* | **0** | **1** | **2** | **3** |
| 3. Konflik disebabkan kerja rumahtangga (*Conflicts over household tasks)* | **0** | **1** | **2** | **3** |
| 4. Masalah atau konflik dengan anak-anak (*Problems or conflicts with children)* | **0** | **1** | **2** | **3** |
| 5.Tekanan dari saudara mara atau ipar/mertua (*Pressure from relatives or in-laws)* | **0** | **1** | **2** | **3** |
| 6. Memperbaiki rumah (*Fixing up of the house)* | **0** | **1** | **2** | **3** |
| 7. Tidak cukup masa untuk bersama keluarga (*Not enough time to spend with family)* | **0** | **1** | **2** | **3** |
| 8. Konflik atau kekecewaan seksual *(Sexual conflict or frustration)* | **0** | **1** | **2** | **3** |
| 9. Persekitaran luar rumah yang merbahaya ataupun penuh dengan tekanan  *(Dangerous or stressful surroundings and neighbourhood)* | **0** | **1** | **2** | **3** |
| 10.Konflik atau kerenggangan dengan kawan Karib atau sudara mara  (*Conflict or falling out with close friend or relative)* | **0** | **1** | **2** | **3** |
| 11.Masalah peribadi yang menyebabkan ketegangan di dalam keluarga  (*Personal problem causing strain in family* | **0** | **1** | **2** | **3** |
| 12.Tiada penjaga anak; susah untuk keluar rumah  (*No babysitters; difficulty getting away from home)* | **0** | **1** | **2** | **3** |

**BAHAGIAN D: TEKANAN TEMPAT KERJA (*PART D: WORKPLACE STRESSOR*)**

**Arahan: Untuk setiap situasi berikut nyatakan kekerapan ia pernah berlaku di unit kerja anda.**

*(For each item indicates how often on your present unit you have found the situations to be stressful)*

| **0** | **1** | **2** | **3** |
| --- | --- | --- | --- |
| Tidak pernah  *(never)* | Jarang-jarang  *(occasionally)* | Kerapkali  *(frequently)* | Sentiasa  *(very frequently)* |

| **A) BEBANAN KERJA** *(WORKLOAD)* | | | | |
| --- | --- | --- | --- | --- |
| 1. Kerosakan alat kelengkapan teknikal/ komputer   *Breakdown of the computer/technical equipment* | **0** | **1** | **2** | **3** |
| 1. Penjadualan kakitangan yang tidak dijangkakan   *Unpredictable staffing and scheduling* | **0** | **1** | **2** | **3** |
| 1. Banyak kerja yang tiada kaitan dengan perawatan diperlukan seperti kerja-kerja perkeranian   *Too many non-nursing tasks required such as clerical work* | **0** | **1** | **2** | **3** |
| 1. Tidak cukup masa untuk memberi sokongan emosi kepada pesakit   *Not enough time to provide emotional support to a patient* | **0** | **1** | **2** | **3** |
| 1. Kekurangan masa untuk menyelesaikan semua kerja-kerja perawatan   *Not enough time to complete all the nursing tasks* | **0** | **1** | **2** | **3** |
| 1. Kekurangan kakitangan untuk mengendalikan unit atau wad tersebut   *Not enough staff to adequately cover the unit* | **0** | **1** | **2** | **3** |
| **B) TENAT DAN KEMATIAN** *(DEATH AND DYING)* | | | | |
| 1. Melaksanakan prosedur yang mendatangkan kesakitan kepada pesakit   *Performing procedures that patients experience as painful* | **0** | **1** | **2** | **3** |
| 1. Kekecewaan disebabkan kegagalan pesakit untuk sembuh   *Feeling helpless in the case of a patient who fails to improve* | **0** | **1** | **2** | **3** |
| 1. Mendengar atau berbual dengan pesakit tentang kematian yang akan ditempuhinya   *Listening or talking to a patient about his/her approaching death* | **0** | **1** | **2** | **3** |
| 1. Kematian seorang pesakit   *The death of a patient* | **0** | **1** | **2** | **3** |
| 1. Kematian seorang pesakit yang telah rapat dengan anda   *The death of a patient with whom you have developed a close relationship* | **0** | **1** | **2** | **3** |
| 1. Ketiadaan doktor bila pesakit meninggal   *Physician not being present when a patient die* | **0** | **1** | **2** | **3** |
| 1. Melihat kesengsaraan pesakit   *Watching a patient suffer* | **0** | **1** | **2** | **3** |
| **C) KURANG PERSEDIAAN** *(INADEQUATE PREPARATION)* | | | | |
| 1. Merasakan tidak bersedia untuk menolong keluarga pesakit dari segi keperluan emosi   *Feeling inadequately prepared to help with the emotional needs of a patient’s family* | **0** | **1** | **2** | **3** |
| 1. Tidak dapat memberi jawapan yang memuaskan kepada pesakit apabila ditanya   *Being asked a question by a patient for which I do not have a satisfactory answer* | **0** | **1** | **2** | **3** |
| 1. Kurang persediaan untuk menolong pesakit dari segi keperluan emosi   *Feeling inadequately prepared to help with the emotional needs of a patient* | **0** | **1** | **2** | **3** |
| **D) KURANG SOKONGAN RAKAN SEJAWAT** *(LACK OF STAFF SUPPORT)* | | | | |
| 1. Kurang peluang untuk bercakap secara terbuka dengan rakan sejawat dari wad lain perihal masalah di wad sendiri   *Lack of an opportunity to talk openly with other unit personnel about problems in the unit* | **0** | **1** | **2** | **3** |
| 1. Kurang peluang berkongsi pengalaman dan perasaan dengan rakan sejawat di wad yang sama   *Lack of an opportunity to share experiences and feelings with other personnel in the unit* | **0** | **1** | **2** | **3** |
| 1. Kurang berpeluang untuk meluahkan rasa tidak puas hati terhadap pesakit kepada rakan sejawat di wad tersebut   *Lack of an opportunity to express to other personnel in the unit my negative feelings*  *toward patients* | **0** | **1** | **2** | **3** |
| **E) TIDAK PASTI DENGAN RAWATAN (***UNCERTAINTY CONCERNING TREATMENT)* | | | | |
| 1. Kurang maklumat tentang keadaan pesakit dari doktor   *Inadequate information from a physician regarding the medical condition of patient* | **0** | **1** | **2** | **3** |
| 1. Doktor memberi arahan yang tidak bersesuaian dengan pesakit   *A physician ordering what appears to be inappropriate treatment for a patient* | **0** | **1** | **2** | **3** |
| 1. Ketiadaan doktor ketika kecemasan berlaku   *A physician not being present in an emergency* | **0** | **1** | **2** | **3** |
| 1. Tidak tahu apa yang sepatutnya diberitahu kepada pesakit dan keluarganya mengenai keadaan pesakit dan rawatannya   *Not knowing what a patient or the family ought to be told about the patient’s condition*  *and its treatment* | **0** | **1** | **2** | **3** |
| 1. Tidak pasti tentang penggunaan dan fungsi peralatan tertentu   *Uncertainty regarding the operation and functioning of specialised equipment* | **0** | **1** | **2** | **3** |
| **F) KONFLIK DENGAN DOKTOR** *(CONFLICT WITH PHYSICIANS)* | | | | |
| 1. Dikritik oleh doctor   *Criticism by a physician* | **0** | **1** | **2** | **3** |
| 1. Konflik dengan doctor   *Conflict with a physician* | **0** | **1** | **2** | **3** |
| 1. Takut melakukan kesilapan di dalam merawat pesakit   *Fear of making a mistake in treating a patient* | **0** | **1** | **2** | **3** |
| 1. Tidak bersetuju berkenaan dengan rawatan pesakit   *Disagreement concerning the treatment of patient* | **0** | **1** | **2** | **3** |
| 1. Terpaksa membuat keputusan megenai pesakit semasa ketiadaan doktor   *Making a decision concerning a patient when the physician is unavailable* | **0** | **1** | **2** | **3** |
| **G) KONFLIK DENGAN RAKAN SEJAWAT** *(CONFLICT WITH OTHER NURSES)* | | | | |
| 1. Konflik dengan supervisor   *Conflict with a supervisor* | **0** | **1** | **2** | **3** |
| 1. Dipinjamkan ke wad lain bila berlaku kekurangan kakitangan di unit tersebut   *Floating to other units that are short-staffed* | **0** | **1** | **2** | **3** |
| 1. Sukar untuk bekerja dengnan rakan sejawat dari wad lain   *Difficulty in working with a particular nurse (or nurses) from other unit* | **0** | **1** | **2** | **3** |
| 1. Dikritik oleh penyelia   *Criticism by a supervisor* | **0** | **1** | **2** | **3** |
| 1. Sukar untuk bekerja dengan rakan-rakan sejawat dari wad yang sama   *Difficulty in working with a particular nurse (or nurses) from the same unit* | **0** | **1** | **2** | **3** |

**BAHAGIAN E: INVENTORI TEKANAN PERIBADI (*PART E: PERSONAL STRESS INVENTORY*)**

**Arahan: Sila nyatakan berapa kerapkah anda mengalami gejala atau masalah berikut.**

*(Indicate how often you have been troubled by the following symptoms or difficulties)*

| 0 | 1 | 2 | 3 |
| --- | --- | --- | --- |
| Tidak Pernah  *(Never)* | Sekali atau Dua Kali Sahaja  *(Once or twice)* | Setiap Minggu  *(Every week)* | Hampir Setiap Hari  *(Nearly everyday)* |

| **A) SISTEM MUSKULOSKELETAL (*MUSCULOSKELETAL SYSTEM)*** | | | | |
| --- | --- | --- | --- | --- |
| 1. Ketegangan otot *(Muscle tension)* | **0** | **1** | **2** | **3** |
| 1. Sakit pinggang *(Back pain)* | **0** | **1** | **2** | **3** |
| 1. Sakit kepala *(Headache)* | **0** | **1** | **2** | **3** |
| 1. Ketap gigi (*Grinding teeth)* | **0** | **1** | **2** | **3** |
| **B) SISTEM GASTROINTESTINAL (*GASTROINTESTINAL SYSTEM)*** | | | | |
| 1. Tidak sedap perut *(Stomach ache or upset)* | **0** | **1** | **2** | **3** |
| 1. Sakit ulu hati (*Heartburn)* | **0** | **1** | **2** | **3** |
| 1. Muntah *(Vomiting)* | **0** | **1** | **2** | **3** |
| 1. Cirit-birit *(Diarrhoea)* | **0** | **1** | **2** | **3** |
| 1. Sembelit *(Constipation)* | **0** | **1** | **2** | **3** |
| 1. Sakit perut *(Abdominal pain)* | **0** | **1** | **2** | **3** |
| **C) SISTEM FIZIKAL LAIN *(OTHER PHYSICAL SYSTEM)*** | | | | |
| 1. Selsema (*Cold or hay fever)* | **0** | **1** | **2** | **3** |
| 1. Sakit dada (*Chest pain)* | **0** | **1** | **2** | **3** |
| 1. Ruam *(Skin rash)* | **0** | **1** | **2** | **3** |
| 1. Mulut kering (*Dry mouth)* | **0** | **1** | **2** | **3** |
| 1. Sakit tekak *(Laryngitis)* | **0** | **1** | **2** | **3** |
| 1. Jantung berdebar-debar (*Palpitation of the heart)* | **0** | **1** | **2** | **3** |
| **D) KETEGANGAN/KERESAHAN *(TENSION/ANXIETY)*** | | | | |
| 1. Menggeletar (*Tremor or trembling)* | **0** | **1** | **2** | **3** |
| 1. Sentak otot *(Twitch or tic)* | **0** | **1** | **2** | **3** |
| 1. Gelap mata (*Dizziness)* | **0** | **1** | **2** | **3** |
| 1. Cemas (*Nervousness)* | **0** | **1** | **2** | **3** |
| 1. Resah (*Anxiety)* | **0** | **1** | **2** | **3** |
| 1. Ketegangan (*Tension and jitters)* | **0** | **1** | **2** | **3** |
| 1. Buntu (*Keyed-up feeling)* | **0** | **1** | **2** | **3** |
| 1. Merisaukan (*Worrying)* | **0** | **1** | **2** | **3** |
| 1. Tidak boleh duduk diam *(Unable to keep still or fidgeting)* | **0** | **1** | **2** | **3** |
| 1. Perasaan takut terhadap sesuatu *(Fear of certain objects or phobias)* | **0** | **1** | **2** | **3** |
| **E) TAHAP TENAGA (*ENERGY LEVEL)*** | | | | |
| 1. Lesu *(Fatigue)* | **0** | **1** | **2** | **3** |
| 1. Kurang bertenaga (*Low energy)* | **0** | **1** | **2** | **3** |
| 1. Sikap tidak ambil peduli (*Apathy or nothing seems important)* | **0** | **1** | **2** | **3** |
| **F) KEMURUNGAN *(DEPRESSION)*** | | | | |
| 1. Sedih/murung (*Depression)* | **0** | **1** | **2** | **3** |
| 1. Ketakutan (*Fearfulness)* | **0** | **1** | **2** | **3** |
| 1. Tiada harapan (*Hopelessness)* | **0** | **1** | **2** | **3** |
| 1. Senang menangis (*Crying easily)* | **0** | **1** | **2** | **3** |
| 1. Menyalahkan diri sendiri (*Highly self-critical)* | **0** | **1** | **2** | **3** |
| 1. Kehampaan (*Frustration)* | **0** | **1** | **2** | **3** |
| **G) TIDUR (*SLEEP)*** | | | | |
| 1. Sukar tidur (*Insomnia)* | **0** | **1** | **2** | **3** |
| 1. Sukar bangun (*Difficulty awakening)* | **0** | **1** | **2** | **3** |
| 1. Mimpi ngeri/mimpi yang mengganggu (*Nightmare or disturbing dream)* | **0** | **1** | **2** | **3** |
| **H) PERHATIAN *(*ATTENTION*)*** | | | | |
| 1. Kemalangan /kecederaan (*Accident or injury)* | **0** | **1** | **2** | **3** |
| 1. Sukar menumpukan perhatian (*Difficulty concentrating)* | **0** | **1** | **2** | **3** |
| 1. Fikiran buntu (*Mind going blank)* | **0** | **1** | **2** | **3** |
| 1. Lupa perkara penting (*Forgetting important information)* |  |  |  |  |
| **I) PEMAKANAN (*EATING)*** | | | | |
| 1. Tiada selera (*Loss of appetite)* | **0** | **1** | **2** | **3** |
| 1. Makan berlebihan (*Overeating or excessive hunger)* | **0** | **1** | **2** | **3** |
| 1. Tiada masa untuk makan (*No time to eat)* | **0** | **1** | **2** | **3** |
| **J) AKTIVITI (*ACTIVITY)*** | | | | |
| 1. Kerja berlebihan (*Overwhelmed by work)* | **0** | **1** | **2** | **3** |
| 1. Tiada masa untuk berehat atau tidak boleh berehat (*No time to relax)* | **0** | **1** | **2** | **3** |
| 1. Tidak dapat menyempurnakan tugas yang ditetapkan (*Unable to meet commitments or complete tasks)* | **0** | **1** | **2** | **3** |
| **K) PERHUBUNGAN (*RELATIONSHIP)*** | | | | |
| 1. Merenggangkan perhubungan (*Withdrawing from relationships)* | **0** | **1** | **2** | **3** |
| 1. Merasa dipergunakan atau mengambil kesempatan (*Feeling victimised or taken advantage of)* | **0** | **1** | **2** | **3** |
| 1. Hilang keinginan atau keseronokan seks (*Loss of sexual interest or pleasure)* | **0** | **1** | **2** | **3** |
